# Enzymatic catalysis treatment method of meat industry wastewater using lacasse

**DOI:** 10.1186/s40201-015-0239-2

**Published:** 2015-12-22

**Authors:** K. Thirugnanasambandham, V. Sivakumar

**Affiliations:** Department of Chemical Engineering, AC Tech Campus, Anna University, Chennai, 600 025 TN India

**Keywords:** Enzymatic catalysis, Meat wastewater, Colour removal, Box-Behnken design, Model development, Process optimization

## Abstract

**Background:**

The process of meat industry produces in a large amount of wastewater that contains high levels of colour and chemical oxygen demand (COD). So they must be pretreated before their discharge into the ecological system.

**Methods:**

In this paper, enzymatic catalysis (EC) was adopted to treat the meat wastewater.

**Results:**

Box-Behnken design (BBD), an experimental design for response surface methodology (RSM), was used to create a set of 29 experimental runs needed for optimizing of the operating conditions. Quadratic regression models with estimated coefficients were developed to describe the colour and COD removals.

**Conclusions:**

The experimental results show that EC could effectively reduce colour (95 %) and COD (86 %) at the optimum conditions of enzyme dose of 110 U/L, incubation time of 100 min, pH of 7 and temperature of 40 °C. RSM could be effectively adopted to optimize the operating multifactors in complex EC process.

## Background

Meat industry is the world’s fastest growing sector due to ever increasing demand of its products. Meat processing industries use approximately 62 Mm^3^/y of fresh water from river and canals [1]. Meat-based products have become an essential part of every day’s life and its high demand has resulted in a large quantity of meat wastewater that needs to be treated in order to protect the environment and aquatic life [2]. The meat wastewater contains higher level of suspended solids and organic materials and these particles cannot be easily separated. For these reasons many attempts have been made to treat meat wastewater using conventional wastewater treatment methods [3]. There are a number of processes available for wastewater treatment such as chemical coagulation, electro coagulation, sedimentation precipitation, ozonation, evaporation, membrane filtration, adsorption, ion-exchange, oxidation and advanced oxidation, incineration, bio-degradation and biological treatment. Moreover, these conventional methods are also usually expensive and treatment efficiency is inadequate because of the large variability of the composition of meat wastewater [4].

Enzymatic catalysis using Laccase (EC) is one of the most practiced technologies extensively used on industrial scale wastewater treatment. Meanwhile, the EC treatment can be simpler and more efficient than the traditional physical-chemical treatments [5]. Laccase has the advantages over conventional chemical or microbial catalysts such as biodegradable, high level of catalytic efficiency, high degree of specificity, easily removed from contaminated streams, easily standardized in commercial preparations and absence of side-reactions [6]. These characteristics provide substantial process energy savings and reduced manufacturing costs. Nevertheless, the efficiency of EC process depends on several factors including the enzyme dose, incubation time, pH and temperature. The optimization of these factors may significantly increase the process efficiency [7].

Traditionally, optimization in wastewater treatment has been carried out by monitoring the manipulate of one factor at a time on an experimental response. While only one factor is changed, others are kept at a constant level. This optimization technique is called one-variable-at-a-time. Its major disadvantage is that it does not include the combined effects among the variables studied [8]. As a consequence, this technique does not depict the complete effects of the parameter on the response. Another disadvantage of the one-factor optimization is the increase in the number of experiments necessary to conduct the research, which leads to an increase of time, man power and operating cost [9]. In order to overcome this problem, nowadays optimization has been carried out by using multivariate statistic techniques. Among the most relevant multivariate techniques used in optimization process wastewater treatment is response surface methodology (RSM). RSM is a collection of mathematical and statistical techniques based on the fit of a polynomial equation to the experimental data, which must portray the performance of a data set with the aim of making statistical previsions. It can be well applied when a response or a set of responses of interest are influenced by several variables. In RSM, Box–Behnken design (BBD) is a statistical technique for designing experiments, building models, evaluating the effects of several factors, and searching optimum conditions for desirable responses. The main advantage of this method of other statistical experimental design methods is the reduced number of experiments trials needed to evaluate multiple parameters and their interactions [10].

An extensive literature survey shows that there is lack of knowledge regarding the optimization of EC paramerets to treat meat industry wastewater using RSM. Hence, in this present study an attempt was made to investigate the optimize the EC process parameters such as enzyme dose, incubation time, pH and temperature on the colour and COD removals from meat industry wastewater using four factors three level Box-Behnken design (BBD). The results will obtain shows the treatment efficiency of EC and its possibility to implement in industrial scale level by analyzing removal efficiencies of colour and COD.

## Methods

### Raw materials and chemicals

Wastewater used in this study was collected from a meat industry located in Erode, Tamilnadu, India and its physico-chemical properties was determined and shown in Table 1. In this present study, analytical reagent grade chemicals such as citrate hydrogen phosphate and di-sodium hydrogen phosphate were used to adjust the pH, which was supplied by Merck chemicals, Chennai. Commercial laccase formulation (DeniLite® IIS; 120 U/g) produced from genetically modified *Aspergillus oryzae* was purchased from local suppliers, Erode.

**Table 1:** Characteristics of meat industry wastewater

Characteristics	Value	Permissible values
pH	5.6	6–8
Colour (CU_s_(Pt-Co)	223	5
COD (mg/l)	4658	500
Turbidity (NTU)	1568	10
Conductivity (mS/cm)	1.78	0.5
BOD (mg/l)	1685	100

### Experimental setup

Jar-test experiments were conducted on meat wastewaters in different graduated glass beakers. A Jar containing 100 ml wastewater were tested at pH range between 5 and 9 with different enzyme dose (80–120 U/L). After each enzyme dose, sample was rapidly mixed at 180 rpm during 3 min and incubated (60–120 min) with different temperature (25–45 °C). After treatment, samples were centrifuged at 10,000 rpm for 15 min and analyzed for colour intensity and for COD.

### Analytical method

Colour, COD, and conductivity were determined according to the standard methods described by American Public Health Association (APHA). Colour measurement was determined with standard dilution multiple method and by comparing absorbance to a calibration curve. Colour removal was determined by monitoring the decrease in the absorbance peak at the maximum wavelength (678 nm). Double beam UV-visible spectrophotometer (Shimadzu UV 1650 PC) was used in all experiments. COD was determined by open reflux method. The sample was refluxed in an acidic medium with a known excess of potassium dichromate (K_2_Cr_2_O_7_) and the remaining dichromate was titrated with ferrous ammonium sulphate (FAS). Turbidity was measured using a turbidity meter (ELIKO, 456) in accordance with standard method via display. The conductivity was determined by conductivity meter (Lil120) via digital display. The removal efficiency (RE) of colur and COD was calculated by using the following equation [11]

1 $$ \mathrm{R}\mathrm{E}=\left(\frac{{\mathrm{c}}_0-{\mathrm{c}}_{\mathrm{e}}}{{\mathrm{c}}_0}\right)\times 100 $$

where, c_0_ and c_e_ is the initial and final concentrations of colour and COD respectively.

### Stastical experimental design

In this present study, Box-Behnken response surface experimental design (BBD) with four factors at three levels was used to optimize and investigate the influence of process variables such as enzyme dose (A), incubation time (B), pH (C) and temperature (D) on enzymatic catalysis process to reduce colour (Y_1_) and COD (Y_2_) from from meat wastewater. Process variables and their ranges (Table 2) were determined based on the preliminary studies. Preliminary studies were carried out using Placket Burmann (PB) design. BBD design consists of 29 experiments with five centre points were designed and the data was analyzed by multiple regression analysis (Sequential sum of squares and model summary statistics) in order to study the ability of various mathematical models to express the enzymatic catalysis process [12].

**Table 2:** Process variables and their ranges

Process variables	Level		
	−1	0	1
A (U/L)	80	100	120
B (min)	60	90	120
C	5	7	9
D (°C)	25	35	45

All the statistical analyses were done with the help of Stat ease Design Expert 8.0.7.1 statistical software package (Stat-Ease Inc., Minneapolis, USA). Then the adequacy of mathematical model was analysed with various statistical analysis such as determination coefficient (R^2^), adjusted determination of coefficient (R_a_^2^), predicted determination of coefficient (R_p_^2^), adequate precision (AP) and coefficient of variation (CV). Then, the individual and combined effects of process parameters on responses were studied by constructing three dimensional (3D) response surface plots from polynomial model [13].

Optimization of process variables for maximum colour and COD was carried out by derringer’s desired function methodology. In this present study, goals of the operating conditions were selected as in a range and the responses goal was selected as maximize. After optimization, adequacy of the model equation for predicting the optimum response value was validated [14]. Triplicate verification experiments were performed under the optimal conditions and the average value of the experiments was compared with the predicted value of the developed model equation [15].

## Results and discussions

In this present study, removal of colour and COD from meat industry wastewater are investigated using enzymatic catalysis method under various operating conditions such as enzyme dose, incubation time, pH and temperature. Four factors with three levels BBD response surface design (BBD) is used to examine and optimize the process variables. BBD experimental design consists of 29 experiments are carried out and the results are shown in Table 3.

**Table 3:** BBD experimental design with results

Run	A	B	C	D	Y_1_	Y_2_
1	120	90	5	35	85.48	69.83
2	100	90	7	35	89.42	79.85
3	120	120	7	35	89.36	79.79
4	120	90	7	25	84.42	74.85
5	80	90	5	35	59.42	55.85
6	120	90	7	45	90.36	80.79
7	100	60	5	35	42.12	39.55
8	100	60	7	25	34.56	24.99
9	100	90	7	35	89.42	79.85
10	80	90	7	25	49.72	48.15
11	100	90	9	25	55.66	50.09
12	100	60	9	35	44.74	39.17
13	100	90	5	45	73.03	67.46
14	100	90	9	45	96.42	86.85
15	100	120	9	35	74.42	67.85
16	80	60	7	35	31.94	22.37
17	100	90	7	35	89.42	79.85
18	100	120	7	45	73.36	63.79
19	100	90	7	35	89.42	79.85
20	100	90	7	35	89.42	79.85
21	80	120	7	35	63.92	50.35
22	100	60	7	45	64.46	54.89
23	100	120	5	35	77.72	66.15
24	100	90	5	25	68.72	59.15
25	80	90	7	45	74.42	66.85
26	120	90	9	35	77.42	67.85
27	120	60	7	35	59.38	49.73
28	100	120	7	25	75.42	65.85
29	80	90	9	35	64.42	50.85

### Mathematical modelling

In order to select the suitable mathematical among various models such as linear, interactive, quadratic and cubic, BBD experimental data is analyzed by multi regression analysis namely the sequential model sum of squares and model summary statistics (Table 4). The obtained results indicates that, linear and interactive (2FI) models were exhibited lower R^2^, adjusted R^2^, predicted R^2^ and also having high *p*-values, when compared with quadratic model. Cubic model was found to be aliased [16]. Therefore the quadratic model ischosen to describe the effects of process variables on the enzymatic catalysis treatment method. Mean while, second-order polynomial equation has been developed by fitting coefficient of process variables (individual and interactive) to the generalized quadratic model and the final model obtained in terms of coded factors are given below

2 $$ \begin{array}{c}{\mathrm{Y}}_1 = 89.42+11.88\mathrm{A}+14.75\mathrm{B}+0.55\mathrm{C}+8.63\mathrm{D}-0.50\mathrm{AB}-3.27\mathrm{AC}-4.69\mathrm{AD}\\ {}-1.48\mathrm{B}\mathrm{C}-7.99\mathrm{B}\mathrm{D}+9.11\mathrm{C}\mathrm{D}-8.05{\mathrm{A}}^2-20.41{\mathrm{B}}^2-9.38{\mathrm{C}}^2-6.76{\mathrm{D}}^2\end{array} $$

3 $$ \begin{array}{c}{\mathrm{Y}}_2 = 79.85+10.70\mathrm{A}+13.59\mathrm{B}+0.39\mathrm{C}+8.13\mathrm{D}+0.52\mathrm{AB}+0.75\mathrm{AC}-3.19\mathrm{AD}\\ {}+0.52\mathrm{B}\mathrm{C}-7.99\mathrm{B}\mathrm{D}+7.11\mathrm{C}\mathrm{D}-8.73{\mathrm{A}}^2-20.33{\mathrm{B}}^2-8.30{\mathrm{C}}^2-5.42{\mathrm{D}}^2\end{array} $$

Where, Y_1_ and Y_2_ are colour and COD removal (%) respectively; A, B, C and D are enzyme dose, incubation time, pH and temperature respectively.

**Table 4:** Sequential model sum of squares and model summary statistics for responses

Source	Sum of squares	DF	Mean Square	*F* Value	Prob > F	Remarks
Sequential model sum of squares for colour removal (%)						
Mean	146045.62	1	146045.62			
Linear	5202.01	4	1300.50	8.09	0.0003	
2FI	727.90	6	121.32	0.70	0.6548	
Quadratic	2998.97	4	749.74	80.57	<0.0001	Suggested
Cubic	129.16	8	16.15	86.70	<0.0001	Aliased
Residual	1.12	6	0.19			
Total	155104.77	29	5348.44			
Sequential model sum of squares for COD removal (%)						
Mean	112009.84	1	112009.84			
Linear	4385.38	4	1096.35	7.14	0.0006	
2FI	502.86	6	83.81	0.47	0.8186	
Quadratic	2934.27	4	733.57	41.62	<0.0001	Suggested
Cubic	191.83	8	23.98	2.62	0.1285	Aliased
Residual	54.92	6	9.15			
Total	120079.10	29	4140.66			
Source	Std.Dev.	R^2^	Adjusted R^2^	Predicted R^2^	PRESS	Remarks
Model summary statistics for colour removal (%)						
Linear	12.6773	0.5742	0.5033	0.4257	5202.4046	
2FI	13.1851	0.6546	0.4627	0.2757	6561.6618	
Quadratic	3.0505	0.9856	0.9712	0.9172	750.4137	Suggested
Cubic	0.4315	0.9999	0.9994	0.9822	160.8996	Aliased
Model summary statistics for COD removal (%)						
Linear	12.3893	0.5435	0.4674	0.3806	4998.1928	
2FI	13.2937	0.6058	0.3868	0.1276	7039.4443	
Quadratic	4.1982	0.9694	0.9388	0.8239	1421.2648	Suggested
Cubic	3.0253	0.9932	0.9682	0.0200	7907.8116	Aliased

### Suitability of developed mathematical models

**Fig. 1:**
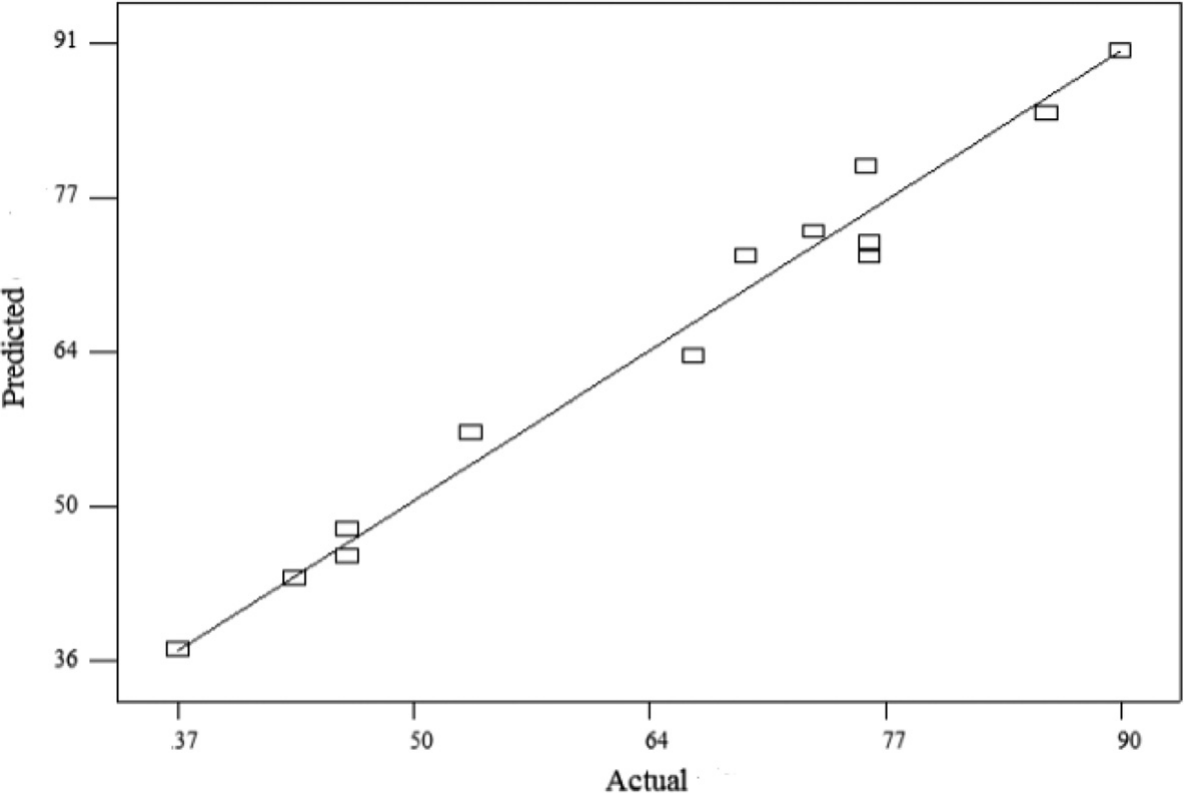
Model adequacy plot

The predicting ability of the developed mathematical model are evaluated by constructing analytical plots (Fig. 1) such as residuals vs run, predicted versus actual plot to find out the connection between predicted and experimental values and also to know the fitness of the model. From the Fig. 1, it is observed that, residuals for the prediction of each response is minimum and it indicated a good adequate agreement between experimental data and the data predicted by model. This result confirms the normal distribution of the observed data and adequacy of the developed model [17]. Statistical significance of the developed mathematical model is examined using pareto analysis of variance (ANOVA) with corresponding *F* and *p*-values of process variables, which is shown in Table 5. The higher model *F* value and lower *p*-values (*p* < 0.0001) confirmed that, the developed model is significant. The goodness of fit of the model is also evaluated by the determination co-efficient of variance (CV), adequate precision (AP) and PRESS, which clearly stated that, the deviations between experimental and predicted values are low and confirms the reliability of the conducted experiments [18].

**Table 5:** ANOVA results for responses

Source	Colour removal (%)		COD removal (%)	
	*F*-value	*P* value	*F*-value	*P* value
Model	68.54	<0.0001	31.70	<0.0001
A	182.05	<0.0001	77.98	<0.0001
B	280.55	<0.0001	125.75	<0.0001
C	0.39	0.5429	0.10	0.7529
D	96.02	<0.0001	44.99	<0.0001
AB	0.11	0.7479	0.06	0.8079
AC	4.58	0.0504	0.13	0.7245
AD	9.45	0.0082	2.31	0.1508
BC	0.94	0.3484	0.06	0.8079
BD	27.44	0.0001	14.49	0.0019
CD	35.69	<0.0001	11.48	0.0044
A^2^	45.15	<0.0001	28.04	0.0001
B^2^	290.24	<0.0001	152.04	<0.0001
C^2^	61.38	<0.0001	25.38	0.0002
D^2^	31.87	<0.0001	10.82	0.0054
C.V. %	4.30		4.85	
PRESS	754.08		596.54	
AP	27.65		31.54	

### Influence of process parameters

**Fig. 2:**
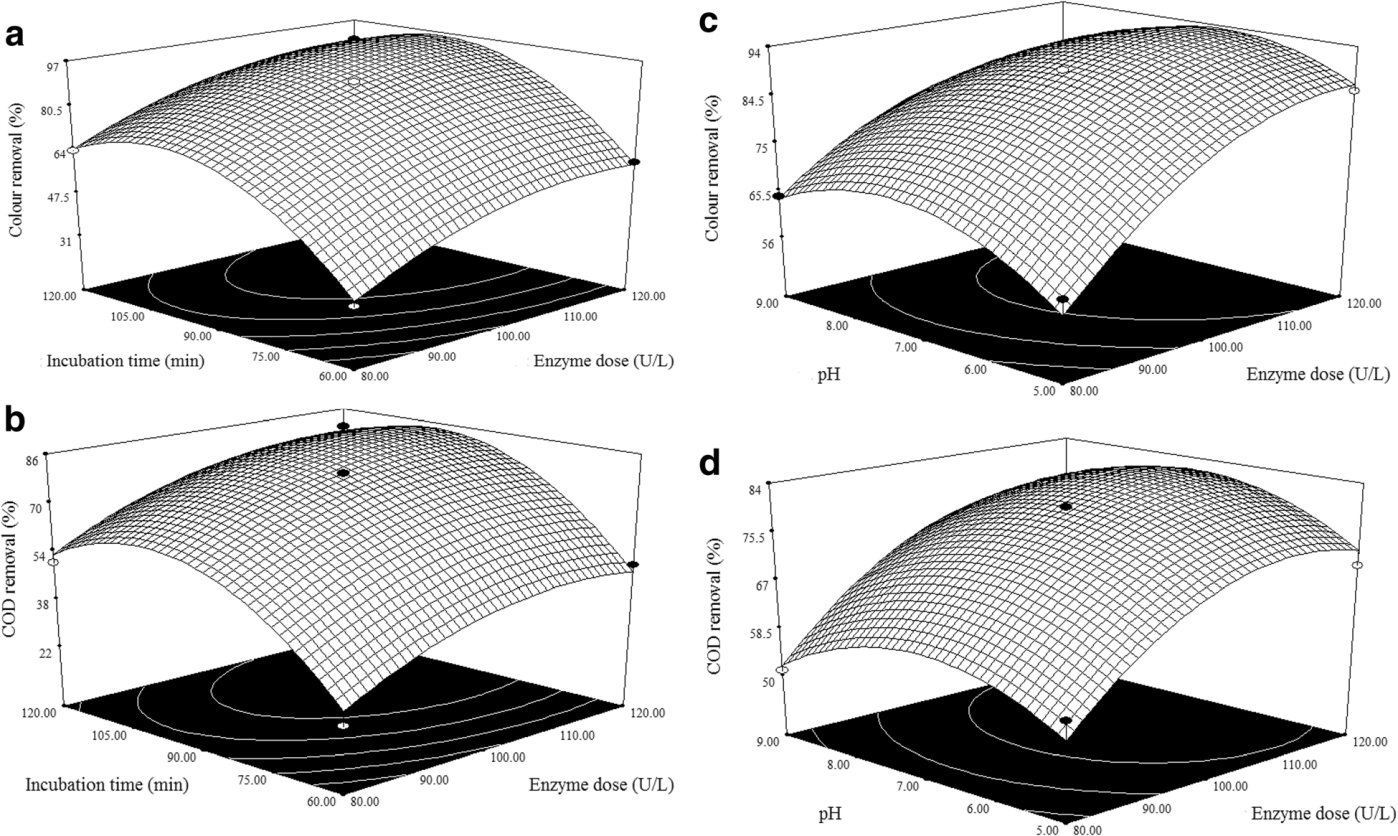
Response surface plots representing the effect of process variables on the responses. **a** and **c**: Colour removal, **b** and **d**: COD removal

Three dimensional (3D) response surface plots are plotted from the developed mathematical models in order to study the individual and combined effect of process variables on the responses to treat meat industry wastewater. In this present study, the model has more than two factors. So, the 3D plots are drawn by maintaining one factor at a constant level (in turn at its central level), whereas the other two factors were varied in their range, which are shown in Fig. 2.

### Effect of enzyme dose

Enzyme dose is one of the crucial parameter, which affects the performance of the enzymatic catalysis for treating meat wastewater significally. So that, experiments were carried out to study the effect of enzyme dose (80, 100 and 120 U/L) over the colour and COD removal and the results are shown in Fig. 2a−c. From the experimental results, it is observed that, the colour and COD removals were increased linearly with increasing enzyme dose upto 110 U/L. This is mainly due to the fact that by increasing the enzyme dose, an increase in the number of active sites takes place. At higher concentration of the enzyme the inhibitors will fall short. More active sites will reduce the colur and COD in the given period of time thus treatment efficiency is enhanced [19]. However, it is noticed that beyond enzyme dose of 110 U/L shows negligible effect on treatment efficiency.

### Effect of incubation time

Incubation time is one of the important factor for the treatment of meat industry wastewater using enzymatic catalysis method. In order to investigate the effect of incubation time, experiments were carried out various incubation time (60, 90 and 120 min) and results are shown in Fig. 2a−d. From the results, it could be found that, the colour and COD removals were increased linearly with increasing incubation time upto 100 min. This is mainly due to the fact that, increase in enzyme dose would increase the reaction kinetic; this happens because free activation centers of the enzyme bind to free substrates thus removal efficiency are increased [20]. Thereafter, there is a negligible effect on the colour and COD removal efficiencies.

### Effect of pH

**Fig. 3:**
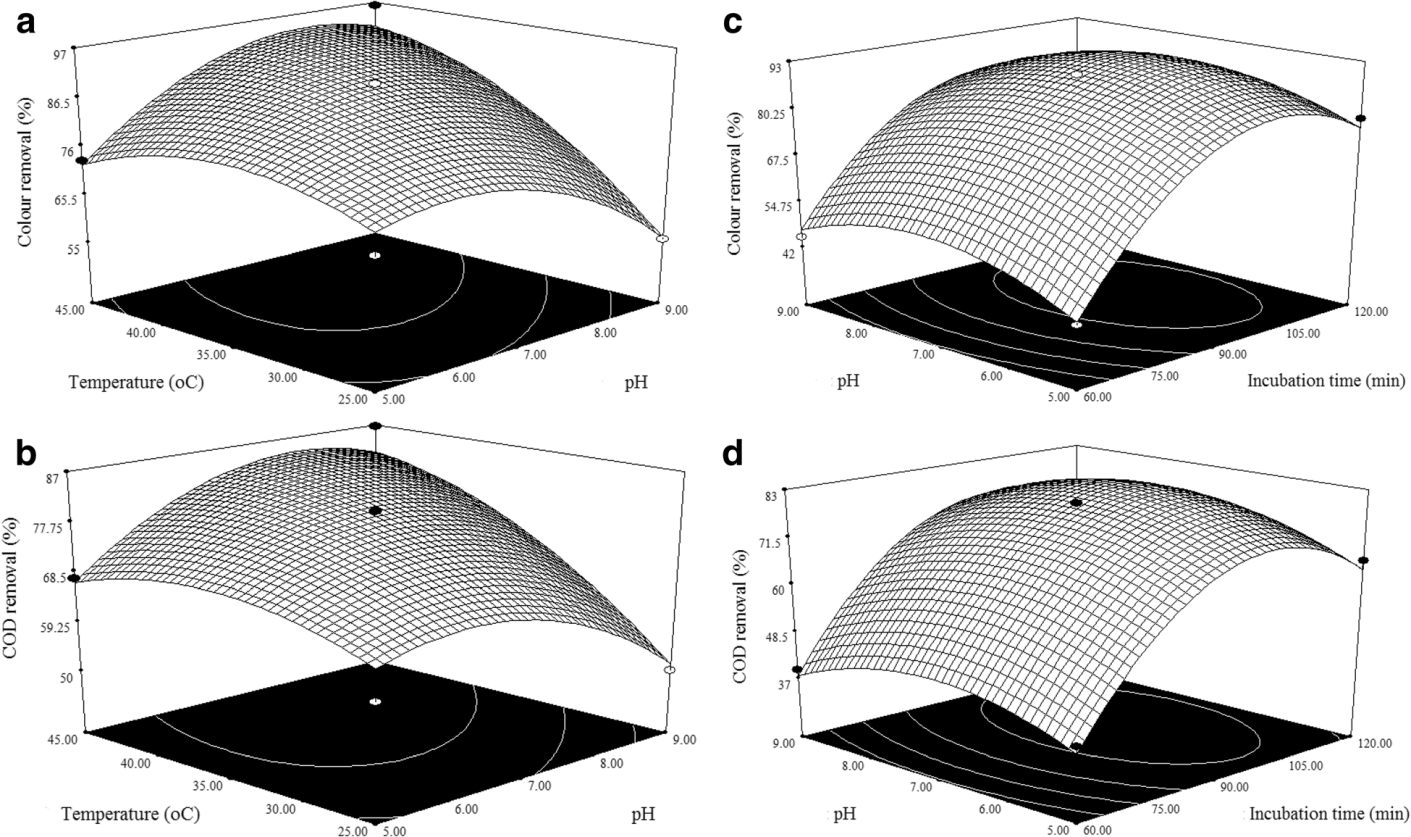
Response surface plots representing the effect of process variables on the responses. **a** and **c**: Colour removal, **b** and **d**: COD removal

The pH is also an important factor influences the treatment of meat wastewater using enzymatic catalysis method. The concentration of hydrogen ions in wastewater affects the enzyme activity. Each enzyme has maximal efficiency under an optimum pH otherwise there will be no enzymatic activity due to denaturation of enzymes. Therefore, in this study influence of pH on the colour and COD removal efficiencies was examined by varying its range (5, 7 and 9) and the results are illustrated in Fig. 3a−c. From the results, it is observed that, colour and COD removal efficiencies was increased with the increasing pH upto 8, due to the increase in the activity of enzyme and enhanced the treatment efficiency [21]. Beyond pH of 8 shows the negligible effect on removal efficiency of colour and COD.

### Effect of temperature

Removal efficiency of colour and COD from meat wastewater using enzymatic catalysis method is highly affectd by temperature and its influence on treatment efficiency is investigated by varying temperature (25, 35 and 45 °C) and the results are depicted in Fig. 3a−d. From the results, it is found that, colour and COD removal efficiencies were increased with the increasing temperature upto 40 °C and it can be explained the fact that, there are distinct temperature ranges under which enzymes operate and there is a specific temperature levels (optimum temperature) in which enzymes have maximum efficiency. Therefore temperature variations affect enzymatic activity and the kinetic of the reactions they catalyze. In addition, enzymes can be denatured under extreme temperatures and loses their catalytic activity [22]. These results indicates the key role of temperature on enzymatic catalysis method process for colour and COD removals.

### Optimization and validation

For optimization, simultaneous optimization of the multiple responses is carried out using Derringer’s desired function methodology in order to find out the optimum operating conditions for maximum removal efficiencies of colour and COD. This numerical optimization technique evaluates a point that maximizes the desirability function and optimum operating conditions were found to be as follows: enzyme dose of 110 U/L, incubation time of 100 min, pH of 7 and temperature of 40 °C. Under these conditions, the experimental results show that EC could effectively reduce the colour (95 %) and COD (86 %). Then, the suitability of optimum conditions for predicting optimum response value is tested based on above mentioned conditions. Triplicate experiments were performed under the optimized conditions and the mean value (95.35, 85.68 % for colour and COD removal respectively) obtained from real experiments, demonstrated the validation of the optimized conditions.

## Conclusion

In this study, BBD was employed to study and optimize the process variables such as enzyme dose, incubation time, pH and temperature on the removal of colour and COD from meat wastewater using enzymatic catalysis method. From the results, it was observed that, all the process variables have significant effects on the treatment efficiency and quadratic model were developed for predicting the responses. Optimum set of the independent variables was obtained by derringer’s desired function methodology in order to find out the maximum colour (95 %) and COD (86 %) removal efficiencies and it was found to be: enzyme dose of 110 U/L, incubation time of 100 min, pH of 7 and temperature of 40 °C. These results indicates that the proposed enzymatic catalysis process is an effective and economically viable method to treat meat industry wastewater.
